# Detection of Gender Differences in Incomplete Revascularization after Coronary Artery Bypass Surgery Varies with Classification Technique

**DOI:** 10.1155/2013/108475

**Published:** 2013-07-08

**Authors:** Sabine Oertelt-Prigione, Friederike Kendel, Martin Kaltenbach, Roland Hetzer, Vera Regitz-Zagrosek, Rufus Baretti

**Affiliations:** ^1^Institute of Gender in Medicine, Charité-Universitätsmedizin Berlin, Luisenstraße 65, 10117 Berlin, Germany; ^2^Institute of Medical Psychology, Charité-Universitätsmedizin Berlin, 10117 Berlin, Germany; ^3^Kardiologisches Centrum Frankfurt, 60316 Frankfurt, Germany; ^4^Deutsches Herzzentrum Berlin, 13353 Berlin, Germany; ^5^Center for Cardiovascular Research (CCR), Charité-Universitätsmedizin Berlin, 10117 Berlin, Germany

## Abstract

*Background*. Incomplete revascularization negatively affects survival after coronary artery bypass surgery (CABG). Since gender and classification technique might impact outcome and reporting, we investigated their effect on revascularization patterns and mortality. *Methods*. A cohort of bypass patients (*N* = 1545, 23% women) was enrolled prospectively. The degree of revascularization was determined as mathematical difference between affected vessels upon diagnosis and number of grafts or the surgeon's rating on the case file. *Results*. Although men displayed more triple-vessel disease, they obtained complete revascularization more frequently than women (85% versus 77%, *P* < 0.001). The two calculation methods identified analogous percentages of incompletely revascularized patients, yet there was only a 50% overlap between the two groups. Mathematically, more women, older patients, and patients with NYHA class III/IV appeared incompletely revascularized, while the surgeons identified more patients undergoing technically challenging procedures. Regardless of the definition, incompleteness was a significant risk factor for mortality in both genders (mathematical calculation: HR 2.62, 95% CI 1.76–3.89, *P* < 0.001; surgeon: HR 2.04, 95% CI 1.35–3.89, *P* = 0.001). *Conclusions*. Given the differences in identification patterns, we advise that the mathematical calculation be performed after-procedure in all patients regardless of the surgeons' rating to uncover additional subjects at increased risk.

## 1. Introduction

The goal of coronary artery bypass graft surgery (CABG) is to improve cardiovascular outcomes by complete revascularization of the diseased vessels. As clinical and anatomical characteristics such as left ventricular ejection fraction (LVEF), previous myocardial infarction, comorbidities, and the calibre of diseased vessels may interact [1], this goal is not always attainable, especially in patients with multivessel disease [2]. The determination of complete revascularization is complicated by the use of different established standards [3]. Customarily, perfusion districts are divided into three main areas, those supplied by the three main coronary branches: the left anterior descending artery, the left circumflex, and the right coronary artery. The “traditional” definition of complete revascularization maintains that at least one bypass is performed in each affected arterial territory [1, 2], while the “functional” definition assumes that all graftable coronary artery segments with significant flow impairment be subjected to bypass [4]. A mathematical “index” given by the ratio between the initially planned number of grafts and the effectively used number has been applied in one study but does not represent the current standard [5].

Several studies have investigated whether incomplete revascularization presents a risk factor for long-term outcome after CABG. Irrespective of the definition used, most studies agree that incomplete revascularization is associated with higher mortality [6, 7]. The association of gender differences with the likelihood of incomplete revascularization and its impact on survival has not been extensively investigated [1, 2, 4, 5, 8–12]. When gender was considered, female patients appear to receive incomplete revascularization more frequently than male patients [13]. Some authors maintain that this is attributable, at least in part, to vessel size [14]; however, this topic remains controversial. While several authors agree that women have a potentially different coronary vasomotor response [15, 16], smaller coronary arteries, and different patterns of atherosclerosis [17, 18], others do not [19]. Furthermore, the size of the vessel does not necessarily correlate with its quality as a target for grafting [20].

As techniques are improving and CABG is performed with increasing frequency, potential inequities in health care provision and performance should be identified and their impact on mortality evaluated. Patients, both women and men, with smaller coronary arteries or other predisposing factors might represent a high-risk group and should be further evaluated pre- and postoperatively. We designed the following study to investigate the interaction between gender and revascularization patterns and to define the impact of incomplete revascularization on 1-year survival in a mixed cohort.

## 2. Materials and Methods

### 2.1. Study Population

Patients undergoing CABG at the Deutsches Herzzentrum (German Heart Institute) Berlin, Department of Cardiothoracic and Vascular Surgery, were identified through daily screening of the admission records. Between January 1, 2005, and July 31, 2008, a total of 1917 patients were approached. Exclusion criteria were (a) inability to read or answer the study questionnaires (e.g., dementia, difficulties with the language), and (b) age below 18 years. Of 1917 patients, 1877 fulfilled all inclusion criteria and 1559 patients provided written informed consent and were thus enrolled in the study. The study protocol was approved by the institutional review board. 

### 2.2. Study Variables

All participants completed a baseline questionnaire 1–3 days before surgery (for complete description see Lehmkuhl et al. [21]). Surgical variables and laboratory values were extracted from case report forms. 

Regardless of the classification, diseased vessels were defined as those affected by coronary artery disease (CAD) as seen by an unequal shape in the two-dimensional angiography regardless of the grade of stenosis >50% and need for revascularization. For the mathematical calculation revascularization status was defined by calculating the difference between the number of diseased vessels and the number of vessels grafted. In this calculation, the fact that left main stem stenosis usually requires two bypasses was accounted for. If the number of diseased vessels corresponded to the number of vessels grafted, the resulting difference was zero. Thus, values of *x* ≥ 1 indicated incomplete revascularization. Results were compared with the dichotomous variable “completeness/incompleteness of revascularization” as reported by the surgeon after surgery on the case report form following the traditional classification. In this case the definition of completeness of revascularization was based on the operational results. 

### 2.3. Statistical Analyses

Of the 1559 patients enrolled in the study, ten patients were excluded due to missing data on the number of grafts received. In four patients, data on diseased vessels were missing, resulting in a final sample of 1545 patients (23.2% women). 

Baseline differences in characteristics between men and women were compared using *t*-tests for continuous variables and *χ* ^2^ square tests for categorical variables. The primary endpoint of the study was 1-year survival. Mortality was defined by the number of deaths of any cause. Mortality data were obtained through hospital records and contacts with either the patient's primary physician or relatives. We estimated the risk of death associated with incomplete revascularization using a univariable Cox proportional hazard model. The associations between the outcome and the features studied were summarized with hazard ratios (HR) and 95% confidence intervals (95% CI). A multiple Cox regression model was fitted to assess whether the influence of incompleteness of revascularization was independent of a set of predefined variables. The statistical analyses were performed using SPSS 18.0 (SPSS Inc., Chicago, IL, USA). For all analyses, an alpha level of *P* < 0.05 was considered significant.

## 3. Results

Men and women differed in a variety of clinical characteristics: women were on average older (70.4 versus 65.8 years; *P* < 0.001) and displaying a more frequent history of hypertension, diabetes, obesity, renal failure, and history of syncope, while men more often had a history of current or former smoking. Women suffered from more advanced heart disease as expressed by a higher percentage of individuals in NYHA class III/IV (14.2% versus 9.4%, *P* = 0.008, Table 1). Intraoperative bypass time was significantly longer in women.

### 3.1. Different Calculations of Incompleteness

According to the mathematical calculation of incompleteness (i.e., the difference between diseased vessels and the number of grafts received) revascularization was incomplete in 16.7% of the entire cohort.

**Figure 1:**
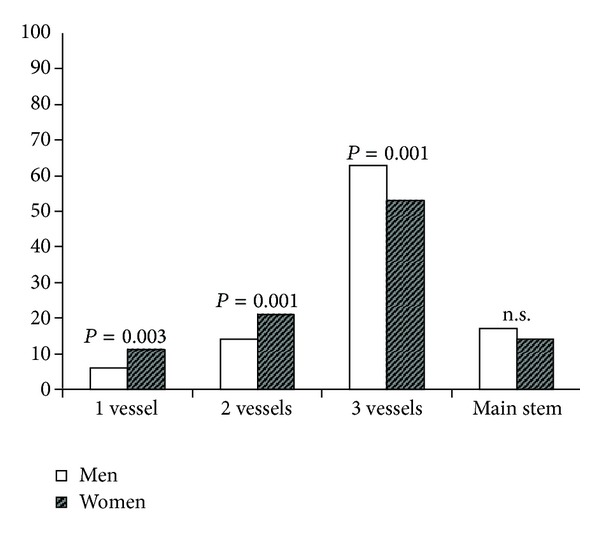
Gender differences in diseased vessels (%). Women more often present with 1-vessel or 2-vessel disease before surgery, whereas men more often present with 3-vessel disease.

**Figure 2:**
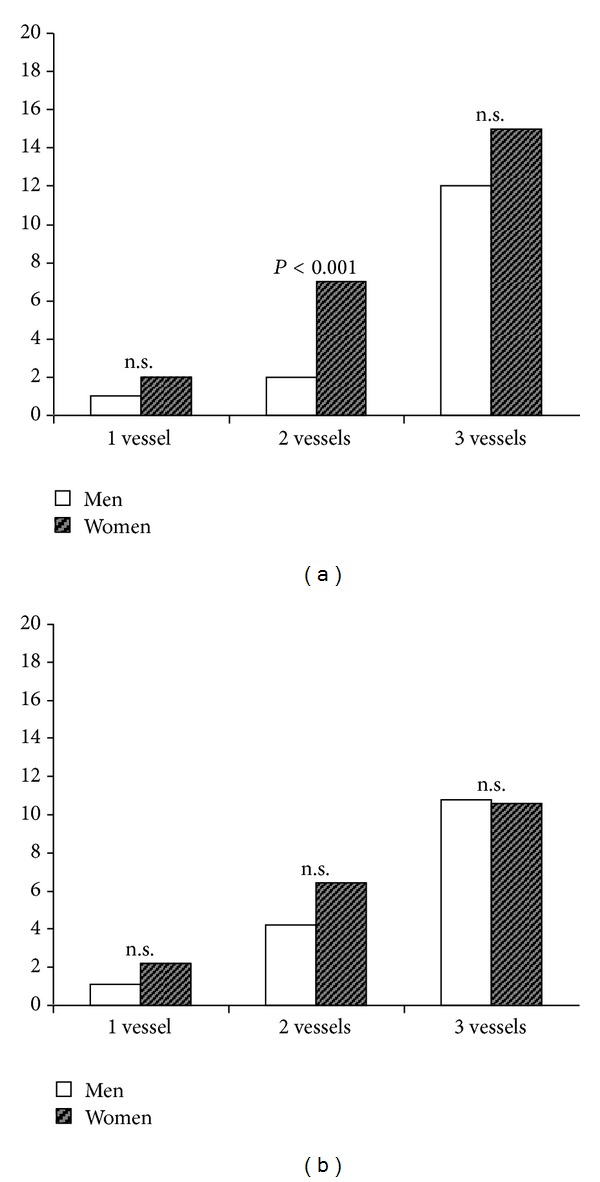
Incomplete revascularization according to the number of diseased vessels (%). According to the mathematical calculation (a), women with double-vessel disease or main stem stenosis more often receive incomplete revascularization than men, whereas the gender differences in single-vessel or triple-vessel disease are not significant. If the surgeons' classification is considered (b), a similar pattern is identified, yet with a relative overestimation of male double-vessel disease and underestimation of female triple-vessel disease.

Female and male patients differed in the number of affected vessels and in revascularization patterns. Women suffered more frequently from single- and double-vessel disease (11.2% and 21.2%, resp.) and men from triple-vessel disease (63.1% males versus 53.4% females, *P* < 0.001, Figure 1). No difference was identified for main stem disease. The increased prevalence of multivessel disease in males did not correlate with worse revascularization patterns. According to the mathematical calculation, 23.5% of the women and 15.2% of the men received incomplete revascularization (*P* < 0.001). As this could potentially be due to different disease patterns in women and men, we separately analyzed incomplete revascularization patterns in relation to the number of diseased vessels. Seven percent of women with double-vessel disease received incomplete revascularization compared to 2% of male patients (*P* < 0.001, Figure 2(a)). No gender differences emerged in the case of triple-vessel disease, with 15% of females receiving incomplete revascularization compared to 12% of males (*P* = 0.08, Figure 2(a)). Using the surgeons' classification, incomplete revascularization was the same regarding single-vessel disease, yet a relative overestimation of the male population with double-vessel disease and a relative underestimation of the women with triple-vessel disease emerged; however, none of these reached statistical significance (Figure 2(b)).

### 3.2. Incomplete Revascularization Is Associated with Increased Mortality

**Figure 3:**
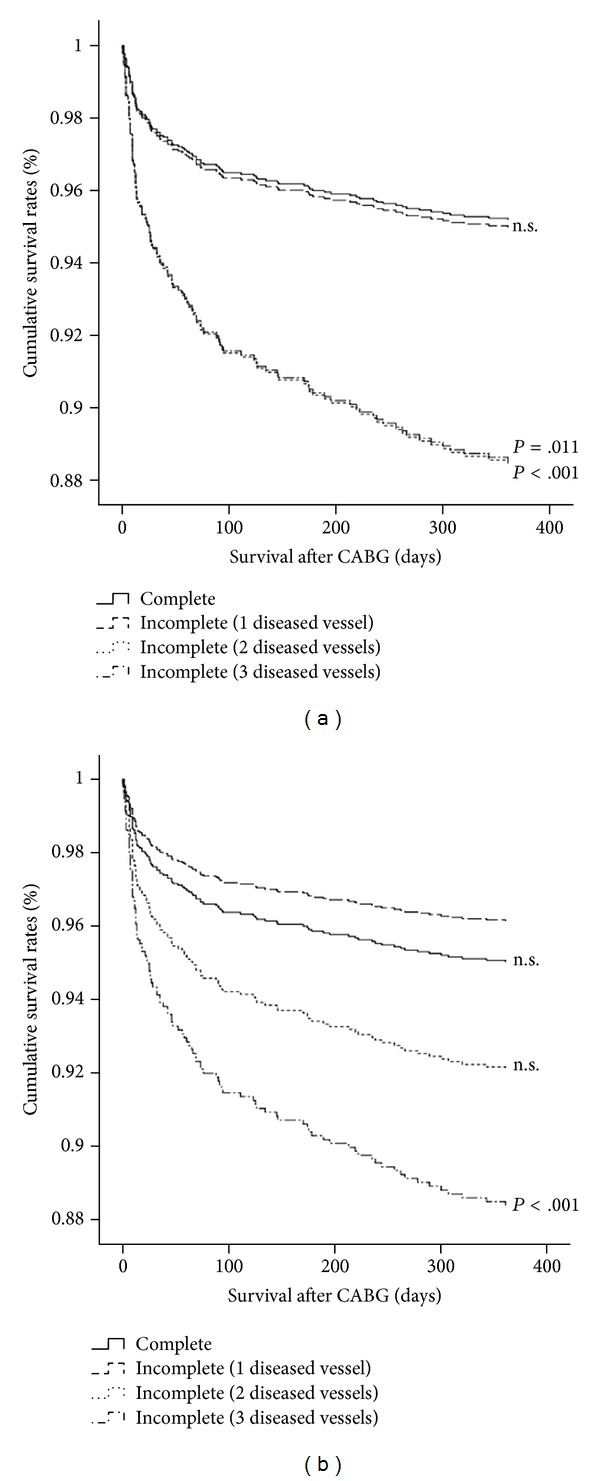
Survival rates after incomplete revascularization according to the number of diseased vessels. According to the mathematical calculation (a), both patients with double- or triple-vessel disease obtaining incomplete revascularization displayed markedly reduced survival rates over 1 year. Using the surgeons' rating (b) the difference reached significance only for patients with triple-vessel disease obtaining incomplete revascularization.

On the basis of our definition, the hazard ratio (HR) for incomplete revascularization was 2.617 (95% CI 1.760–3.890; *P* < 0.001). Incomplete revascularization significantly increased 1-year mortality in patients with double- and triple-vessel disease if the mathematical calculation is used (Figure 3(a)). The association affected both genders. Using the surgeons' rating only the mortality of patients with triple-vessel disease appeared significantly increased compared to fully revascularized patients (Figure 3(b)). After adjustment for clinical variables and comorbidities, incomplete revascularization still remained an independent risk factor for mortality (HR = 2.157, 95% CI 1.439–3.234; *P* < 0.001, Table 2). In addition to incomplete revascularization, age, LVEF, renal dysfunction, and urgency of procedure were significant predictors for 1-year mortality in the multiple Cox regression analysis (Table 2). Interestingly, in post hoc analyses to define the relationship between incomplete revascularization and postoperative complications (postoperative myocardial infarction, low cardiac output, pericardial effusion, postoperative cerebral event, respiratory insufficiency, infection, reanimation, and rethoracotomy), the mathematical calculation showed significant relationship of incompleteness with low cardiac output (*P* = 0.006), respiratory insufficiency (*P* = 0.024), infection (*P* = 0.027), and a trend towards the need for reanimation (*P* = 0.081). Using the surgeon's rating, only the relationship with respiratory insufficiency was significant (*P* = 0.017).

### 3.3. Different Definitions of Incomplete Revascularization Identify Different Subsets of Patients

We sought to compare the results obtained using the mathematical definition with the surgeon's rating of incomplete revascularization. While both definitions identified 16.7% of the sample as incompletely revascularized, the definitions agreed on only 50% of individual patients of the sample (Table 3). Compared to the mathematical calculation, the surgeon's rating was associated with a relative underestimation of the mortality risk with an HR estimate of 2.044 (95% CI 1.347–3.890, *P* = 0.001). The population of patients identified with the mathematical index differed from the ones identified by the surgeons. Patients belonging to this group were more frequently older than 70 years (39.2% versus 22.9%, *P* = 0.005), female (32.3% versus 21.4%, *P* = 0.05), and classified as in NYHA III/IV (15.4% versus 6.9%, *P* = 0.031), while the ones identified by the surgeon's rating included more men (78.6% versus 67.7%, *P* = 0.05) and a significantly larger proportion of patients receiving three or more grafts (75% versus 0%, *P* < 0.001). Since patients were classified as affected by single-, double- or triple-vessel disease, the mathematical calculation will label every patient receiving more than three grafts as completely revascularized. No difference could be detected in procedure duration.

## 4. Discussion

In accordance with previous studies describing the adverse effect of incomplete revascularization [2, 5, 11], the key results of our analyses indicate that (1) incomplete revascularization is an independent risk factor for 1-year mortality; that (2) although women appear to suffer more frequently from less extensive disease, complete revascularization is still achieved significantly more often in the male population; and that (3) a mathematical calculation of incomplete revascularization identified a larger number of women, older patients, and more clinically advanced patients, as opposed to the surgeon's rating which more frequently included males and subjects receiving three or more grafts, which results in more technically complex procedures. 

The role of incomplete revascularization as a risk factor for postoperative mortality is in accordance with previous findings [6, 7]. As multiple reports from our group and others have identified that women die more often after bypass surgery [21–23], we postulate that incomplete revascularization appears to be one of several explanatory factors for the increased risk of mortality in women. 

Women's coronary arteries have been reported to display a different pattern of vascular calcification and reactivity to acetylcholine stimulation compared to men, which is not necessarily visible upon angiography [15, 16] and could potentially lead to over, and underestimation of disease. Female patients in our study population were older, affected by more advanced disease according to the NYHA classification, and displayed a higher frequency of comorbidities and risk factors, yet the number of diseased vessels identified preoperatively was lower than that in the male population. This might be partially explained by taking into account some of the described anatomical and functional differences [15]. However, preoperative classification might have potentially underestimated disease burden in these patients, leading to a less favorable outcome after the procedure.

The variability described might also explain some of the classification discrepancies between the two methods. First, coronary vessels affected by atherosclerosis and irregular wall structure are classified as diseased vessels. Without significant stenosis and intraluminal flow limitation they will not be bypassed due to competing flows and the risk of early graft occlusion. During the first year after CAGB a possible progression of arteriosclerosis at this site could cause ischemia and increased mortality. Second, smaller vessels with a concentric type of atherosclerosis in a longer segment (diffuse small-vessel disease) might not be identified as diseased vessels or, due to a missing poststenotic landing zone for the bypass anastomosis, might remain unrevascularized leading to ischemic risk. Third, vessels subject to altered dilator response could have been classified as diseased upon imaging but perceived differently by the surgeon. Detection—visual and palpatory—of this type of pattern might prove difficult, if not impossible, hampering the estimation of vessel patency after the procedure. In fact, we noticed relative over- and underestimates of incomplete revascularization in the double- and triple-vessel disease patients according to gender. A purely mathematical calculation performed after the procedure might be helpful in the prediction of these cases. The fact that more women are identified by the mathematical index supports the assumption that these anatomical peculiarities are more common in the female population.

Incomplete revascularization after the most technically challenging procedures, that is, those characterized by the implantation of multiple bypasses, is most effectively judged by the surgeon. Intuitively, evaluation of the target vessel, its patency rate, and flow characteristics after the implantation of multiple grafts in severely diseased patients can be best performed by direct inspection. Risk factors for early graft occlusion are also best identified during the procedure, leading to the decision to avoid surgical revascularization. Interestingly, these patients are more frequently male, raising the question whether direct identification of incomplete revascularization might be facilitated in male patients, possibly due to anatomical factors, that is, most likely vessel size. This underscores that, while the mathematical calculation certainly raises important questions about risk underestimation in a specific subgroup of patients, it cannot substitute the surgeon's judgement, especially in complicated cases. 

It should be considered that patients in the current study were enrolled in a highly specialized tertiary referral centre, leading to the inclusion of a disproportionately large number of patients with complicated and advanced disease. Furthermore, the proposed mathematical index, which might aid the identification of a subgroup of patients at risk, can only be performed post hoc. This allows for possible prediction of an increased risk of postoperative complications but cannot facilitate intraoperative identification of these patients and consequent decision-making.

In conclusion, incomplete revascularization appears as a significant risk factor for 1-year mortality in the patient population undergoing CABG surgery. While age and disease severity are nonmodifiable risk factors that do not intrinsically associate with one gender, incomplete revascularization could be potentially reduced and disproportionately affects the female patient population. Completeness of revascularization should be a major aim in patients of both genders, whenever technically feasible. Anatomical differences might affect misclassification of disease severity and represent an area where research is needed. Critical decision-making tools, such as improved imaging techniques, possibly with gender-specific evaluation thresholds, as well as improved pre- or intrasurgical decision algorithms could identify patients at increased risk, prevent some of the problems described, and potentially reduce one-year mortality. One interesting approach is the generalized performance of fractional flow reserve (FFR) measurements in all patients before undergoing CABG. A recent study by Kim et al. investigated potential sex differences in FFR measurements and their effect on mortality [24]. For both women and men, FFR measurement improved survival. Furthermore, the study highlighted a potential overestimation of stenosis by angiography compared to FFR in women. While this finding is descriptive and the authors' themselves maintain that differences in vessel size and potentially lower peak flows due to increased incidence of microvascular dysfunction could play a role, it is, nonetheless, a very promising step in the right direction.

## Figures and Tables

**Table 1:** Baseline characteristics of the study population.

Baseline variables	Total (*N* = 1545)	Men (*n* = 1187)	Women (*n* = 358)	*P* value
Age (yrs), M ± SD	669 (9.0)	65.8 ± 8.6	70.4 ± 9.4	<0.001
History of PCI or CABG (%)	496 (32.1)	381 (32.1)	115 (32.1)	0.993
Hypertension (%)	1352 (87.5)	1015 (85.5)	337 (94.1)	<0.001
Diabetes (%)	548 (35.5)	403 (34.0)	145 (40.5)	0.023
Hypercholesterolemia (%)	1208 (78.2)	925 (77.9)	283 (79.1)	0.652
Smoking (former or current) (%)	1093 (70.7)	917 (77.3)	176 (49.2)	<0.001
Renal failure (%)	428 (27.7)	262 (22.1)	166 (46.4)	<0.001
BMI >30 (%)	417 (27.0)	303 (25.5)	114 (31.8)	0.018
History of syncope (%)	248 (16.1)	166 (14.0)	82 (22.9)	<0.001
History of cerebrovascular event (%)	180 (11.7)	137 (11.5)	43 (12.0)	0.808
NYHA class III/IV (%)	162 (10.5)	111 (9.4)	51 (14.2)	0.008
LVEF <45 (%)	311 (20.1)	247 (20.8)	64 (17.9)	0.225
COPD (%)	202 (13.1)	150 (12.6)	52 (14.5)	0.200
Urgent/emergency procedure (%)	386 (25.0)	304 (25.6)	82 (22.9)	0.300
Bypass time >180 min., M ± SD	95 (6.1)	64 (5.4)	31 (8.7)	0.024
Off pump (%)	55 (3.6)	44 (3.7)	11 (3.1)	0.570

PCI: percutaneous coronary intervention; BMI: body mass index; LVEF: left ventricular ejection fraction; COPD: chronic obstructive pulmonary disease.

**Table 2:** Incomplete revascularization is associated with 1-year mortality after adjustment for multiple risk factors. Results from multiple Cox regression analysis.

	HR	95% CI	*P* value
Age	1.051	1.026–1.076	<0.001
Gender	1.526	0.981–2.374	0.061
LVEF	3.339	2.249–4.957	<0.001
Smoking	1.357	0.857–2.151	0.193
Diabetes	0.932	0.628–1.383	0.725
Hypertension	1.234	0.689–2.208	0.479
Renal dysfunction	1.679	1.124–2.506	0.011
COPD	1.511	0.931–2.452	0.095
Urgency of procedure	1.859	1.258–2.747	0.002
Incomplete revascularization	2.157	1.439–3.234	<0.001

CI: confidence interval; HR: hazard ratio.

**Table 3:** Clinical variables and their frequencies within the incompletely revascularized patient populations identified by two different definitions.

Variable	Mathematical calculation (*n* = 130)	Surgeon's rating (*n* = 131)	*P* value
Age >70 years	51 (39.2%)	30 (22.9%)	0.005
Female gender	42 (32.3%)	28 (21.4%)	0.051
NYHA III/IV	20 (15.4%)	9 (6.9%)	0.031
Bypass time >180 min.	9 (6.9%)	10 (7.6%)	1.00
Number of grafts >3	0 (0%)	98 (74.8%)	<0.001

## Abbreviations

CABG: Coronary artery bypass grafting
LVEF: Left ventricular ejection fraction
COPD: Chronic obstructive pulmonary disease
PCI: Percutaneous coronary intervention
BMI: Body mass index
HR: Hazard ratio
CI: Confidence interval.
